# Easily Tunable Membrane Thickness of Microcapsules by Using a Coordination Assembly on the Liquid-Liquid Interface

**DOI:** 10.3389/fchem.2018.00387

**Published:** 2018-09-07

**Authors:** Bei-xing Li, Xiao-xu Li, Yang Liu, Da-xia Zhang, Jin Lin, Wei Mu, Feng Liu

**Affiliations:** ^1^Key Laboratory of Pesticide Toxicology and Application Technique, College of Plant Protection, Shandong Agricultural University, Tai'an, China; ^2^Research Center of Pesticide Environmental Toxicology, Shandong Agricultural University, Tai'an, China

**Keywords:** microcapsule, polyphenol, metal ion, deposition, coordination assembly, membrane thickness

## Abstract

A model solvent, 1,3,5-trimethylbenzene, was encapsulated using coordination assembly between metal ions and tannic acid to reveal the deposition of coordination complexes on the liquid-liquid interface. The deposition was confirmed by zeta potential, energy dispersive spectroscopy and X-ray photoelectron spectroscopy. Scanning electron microscopy and transmission electron microscopy were integrated to characterize the microcapsules (MCs). According to atomic force microscopy height analysis, membrane thickness of the MCs increased linearly with sequential deposition. For MCs prepared using the Fe^3+^-TA system, the average membrane thicknesses of MCs prepared with 2, 4, 6, and 8 deposition cycles were determined as 31.3 ± 4.6, 92.4 ± 15.0, 175.4 ± 22.1, and 254.8 ± 24.0 nm, respectively. Dissolution test showed that the release profiles of all the four tested MCs followed Higuchi kinetics. Membrane thicknesses of MCs prepared using the Ca^2+^-TA system were much smaller. We can easily tune the membrane thickness of the MCs by adjusting metal ions or deposition cycles according to the application requirements. The convenient tunability of the membrane thickness can enable an extensive use of this coordination assembly strategy in a broad range of applications.

## Introduction

Microencapsulation technology is a promising approach that has been widely reported to protect sensitive core materials, including but not limited to chemicals and living biomaterials (Parthasarathy and Martin, 1994; Anderson and Shive, 2012; Tong et al., 2012; Li B.-X. et al., 2017). The past few decades have seen significant advances of microencapsulation in drug delivery (Wang et al., 2006; De Koker et al., 2012; Simoes et al., 2015; Jia et al., 2017; Li H. et al., 2017), material industry (White et al., 2001; Su and Schlangen, 2012; Jamekhorshid et al., 2014), biomaterials (Parthasarathy and Martin, 1994; Zhi and Haynie, 2006; Kurayama et al., 2012; Ekanem et al., 2017; Zhao et al., 2017), agrochemicals (Li et al., 2016, 2018; Liu et al., 2016; Liang et al., 2017), food industry (Xu et al., 2013), and other fields. There are numerous encapsulation methods focusing on *in situ* polymerization (Su et al., 2013; Zuo et al., 2014), interfacial polymerization (Wagh et al., 2009), spray drying (Zhang and Zhong, 2013), solvent evaporation (Lee et al., 2012) and so on. However, these methods show some drawbacks in terms of financial cost, time cost, simplicity or environmental compatibility, as listed in Table 1. Thus, more environmentally friendly and versatile methods are urgently required.

**Table 1:** Advantages and disadvantages of various encapsulation methods.

**Encapsulation method**	**Advantage**	**Disadvantage**
*In situ* polymerization	Inexpensive wall materials and simple manufacturing equipment (Sukhorukov et al., 2001)	Highly complex procedure (Fan and Zhou, 2010); An important precursor, formaldehyde, has a pungent smell and high toxicity for higher animal life (Cassee et al., 1996; Zuev et al., 2013)
Interfacial polymerization	Simple process and lower emphasis on monomer purity (Wagh et al., 2009; Zhang et al., 2018a)	Time-consuming; high cost of polymer monomer (Li et al., 2013; Zhang et al., 2016)
Spray drying	Simple procedure (Wang et al., 2016)	High energy consumption (Aghbashlo et al., 2012) Low encapsulation efficiency (Varona et al., 2013)
Solvent e vaporation	Simple procedure (Fan et al., 2014)	Low drug-loading efficiency (Lee et al., 2012)

In recent years, coordination complexes utilizing metal ions and natural products have attracted extensive interest from the scientific community due to their high environmental compatibility (Bentley and Payne, 2013; Ejima et al., 2013; Guo et al., 2014; Li et al., 2016; Rahim et al., 2016). These materials can be assembled onto planar or particulate substrates with a range of functionalities, including enhanced mechanical stability selection, permeability, and stimuli-responsiveness (Rahim et al., 2014; Ping et al., 2015; Ejima et al., 2016; Richardson et al., 2016). However, few reports had explored the deposition of such materials on a liquid-liquid interface. Since the interfacial properties of the liquid-liquid interface and the solid-liquid interface differ significantly, the deposition process of coordination complexes may be very different. Therefore, urgent exploration is required to characterize the deposition process based on the liquid-liquid interface.

Microcapsules (MCs) prepared with these polymers always release slowly in target sites because the polymers can hardly degrade in water, soil, or air (Zhang et al., 2016). However, in addition to long-term effectiveness, a rapid efficacy of core materials is also required in many situations, such as the rapid activity in emergency medicine and rapid insecticidal efficacy in agriculture. Moreover, the residual and other unforeseeable risks of core materials in the environment also increase due to the slow release of core materials after the encapsulation (Li et al., 2014). Release profiles of MCs in conventional systems have often been regulated by the membrane thickness, particle size, pore size, membrane permeability or stimuli-gated channels (Siepmann and Siepmann, 2012; Jeong et al., 2014; Zhang et al., 2014, 2018b; Shi et al., 2016; Cui et al., 2017; Huang et al., 2017). The fabrication of wrinkled MCs is another important strategy for increasing the surface area of the membrane, but little progress was observed in terms of the release rate (Ina et al., 2016). MCs prepared with coordination complexes were reported to degrade faster and the release of core materials was pH-responsive (Ejima et al., 2013). Thus, we would like to provide strategies for achieving the tunable membrane thickness of MCs by using coordination assembly.

As proof, we report the formation of metal-polyphenol networks on the liquid-liquid interface using 1,3,5-trimethylbenzene as a model core material. The deposition was confirmed by zeta potential, EDS and XPS. SEM, TEM, and AFM were also integrated to characterize the MCs. We have thus provided convenient strategies for obtaining a tunable membrane thickness of MCs by using coordination assembly.

## Materials and methods

### Reagents

The solvent 1,3,5-trimethylbenzene (purity of 97%), and the wall materials tannic acid (TA) (C_76_H_52_O_46_, AR), iron (III) chloride hexahydrate (FeCl_3_·6H_2_O, ACS), and calcium chloride anhydrous (CaCl_2_, ACS) were all purchased from Aladdin Reagent Co. Ltd., (Shanghai, China). Calcium lignosulfonate was purchased from Yanbian Chenming Paper Industry Co., Ltd., (Jilin, China). Sodium lignosulfonate with the molecular weight of 10,000–12,000, and sulfonation degree of 0.85 mmol g^−1^ was purchased from MeadWestvaco Holding Co. Ltd., (Shanghai, China). Distilled water was used throughout the study.

### Preparation of solvent-loaded MCs

The standard procedure used in this work is as follows: in the first step, 30.93 g of 1,3,5-trimethylbenzene was accurately weighed as the organic phase. Then 1 g of surfactant was dissolved in 40 g of water to obtain the aqueous continuous phase. After pouring the organic phase into the aqueous continuous phase, homogenization at 10,000 rmin^−1^ was implemented with a homogenizer (BRT-25w, Shanghai BRT Equipment Technology CO., Ltd., Shanghai, China) to fabricate a fine emulsion. Subsequently, the mixture was transferred to magnetic stirring at 1,000 r min^−1^. Solutions of FeCl_3_·6H_2_O (or CaCl_2_ for the Ca^2+^-TA system) and TA were then added to deposit on the oil-water interface. Finally, we obtained solvent-loaded MCs after reaction for another 5 min.

### Characterization of solvent-loaded MCs

Surface zeta potentials of the samples were measured using a Zetasizer Nano ZS90 (Malvern Instruments, UK). Scanning electron microscopy (SEM) images were obtained using a Quanta 250 SEM instrument (FEI, USA) operated at the acceleration voltage of 20 kV. Energy dispersive spectroscopy data were obtained using an X-Max EDS system (Oxford Instruments, UK). Transmission electron microscopy (TEM) images were acquired using a JEM-2100 TEM (JEOL Ltd., Japan) with an operational voltage of 200 kV. Atomic force microscopy (AFM) experiments were conducted using a Bruker Multimode 8 scanning probe microscope (Bruker Corporation, German). Tapping mode was used in AFM scans, and the system was equipped with a recommended RTESP probe. Prior to the AFM measurements, core materials encapsulated in MCs were dissolved in excessive methanol solution to yield hollow MCs. Then, hollow MCs were allowed to air-dry for 48 h on mica discs prior to height analysis (Ted Pella, Inc., California, USA). Membrane thicknesses of the air-dried MCs were analyzed using Nanoscope Analysis image processing software (v1.40, Bruker Corporation). Release profiles of the MCs were measured according to previously reported protocols (Cui et al., 2017).

## Results and discussion

### Preparation and characterization of MCs prepared using Fe^3+^ and TA

To describe the deposition of metal-polyphenol networks on the liquid-liquid interface, we used 1,3,5-trimethylbenzene as a model core material because it is a common hydrophobic solvent. Successful deposition on 1,3,5-trimethylbenzene would provide a reference for various capsule suspensions used in agrochemicals and food chemistry, where hydrophobic chemicals are always encapsulated. Unlike coating on planar or particulate substrates (Ejima et al., 2013), we need a suitable emulsifier to fabricate a fine emulsion with a certain size distribution before the deposition process. Anionic surfactants, such as calcium lignosulfonate and sodium lignosulfonate, showed favorable emulsification. Moreover, the coordination complexes could deposit on the liquid-liquid interface when using calcium lignosulfonate as the surfactant.

We have previously reported that when Fe^3+^ was added first, the final pyraclostrobin-loaded MCs tend to be relatively smooth, whereas MCs are prone to agglutinate when TA was first added (Li B.-X. et al., 2017). Therefore, we used the addition sequence of Fe^3+^ + TA + Fe^3+^ + TA + Fe^3+^…in the current study. When 1,3,5-trimethylbenzene and calcium lignosulfonate were used, we obtained a gray and white fine emulsions. Thereafter, the emulsion became gray after the addition of 1 ml of 0.12 mol l^−1^ Fe^3+^ solution. Meanwhile, addition of Fe^3+^ shifted the surface zeta potential (ζ) from −45.4 ± 3.6 mV to −35.1 ± 3.2 mV. Subsequently, the mixture turned blue and then black after the gradual addition of all of the 2 ml TA solution (0.03 mol l^−1^). Finally, we successfully fabricated solvent-loaded MCs after the sample was centrifuged at 2,000 r min^−1^ for 1 min, removed the supernatant and added distilled water to redisperse the remained MCs. The above process was defined as one cycle. Next, we had investigated whether multilayered membrane could be deposited on the liquid phase by repeating the standard process. Another addition of Fe^3+^ solution (1 ml, 0.12 mol l^−1^) made it 1.5 cycles and shifted ζ from −42.2 ± 4.1 mV to −37.3 ± 3.2 mV. Two cycles finished as soon as 2 ml of TA solution (0.03 mol l^−1^) was added, centrifuged and redispersed. As depicted in Figure S1, surface ζ varied greatly with deposition cycles although the values were always negative. Absolute values of zeta potential (|ζ|) increased as soon as TA was added at 1, 2, 3, 4, 5, 6, 7, and 8 deposition cycles (compared with the former addition of Fe^3+^), which was attributed to the acidic nature of the galloyl groups in TA. However, overall, the gradual decreases in |ζ| could be apparently observed with the deposition cycles; |ζ| decreased to approximately 25 mV after 5 cycles, significantly lower than that of the first few cycles (approximately 36 mV). In the fundamental theory of collochemistry, lower |ζ| was demonstrated to yield worse colloidal stability (Feng et al., 2010). Therefore, we deduced that the system became relatively unstable with the increasing deposition cycles.

The presence of TA and Fe in the membrane was then confirmed by energy dispersive spectroscopy (EDS). The EDS spectra of representative 1,3,5-trimethylbenzene-loaded MCs confirmed the existence of C, O, S, Cl, Ca and Fe (Figure S2A), and element composition is described in Table S1. The C and O mainly originated from tannic acid, whereas S and Ca originated from the calcium lignosulfonate molecules. The Cl and Fe elements were derived from the addition of the FeCl_3_ solution. However, X-ray photoelectron spectroscopy (XPS) only confirmed the presence of O, C and Cl (Figure S2B). We supposed that the signal of Fe2p was masked due to the complicated composition of the MCs and smaller penetration depth of XPS in comparison with EDS. Ejima et al. (2013) also indicated that the Fe2p signal was weak in a similar system.

**Figure 1:**
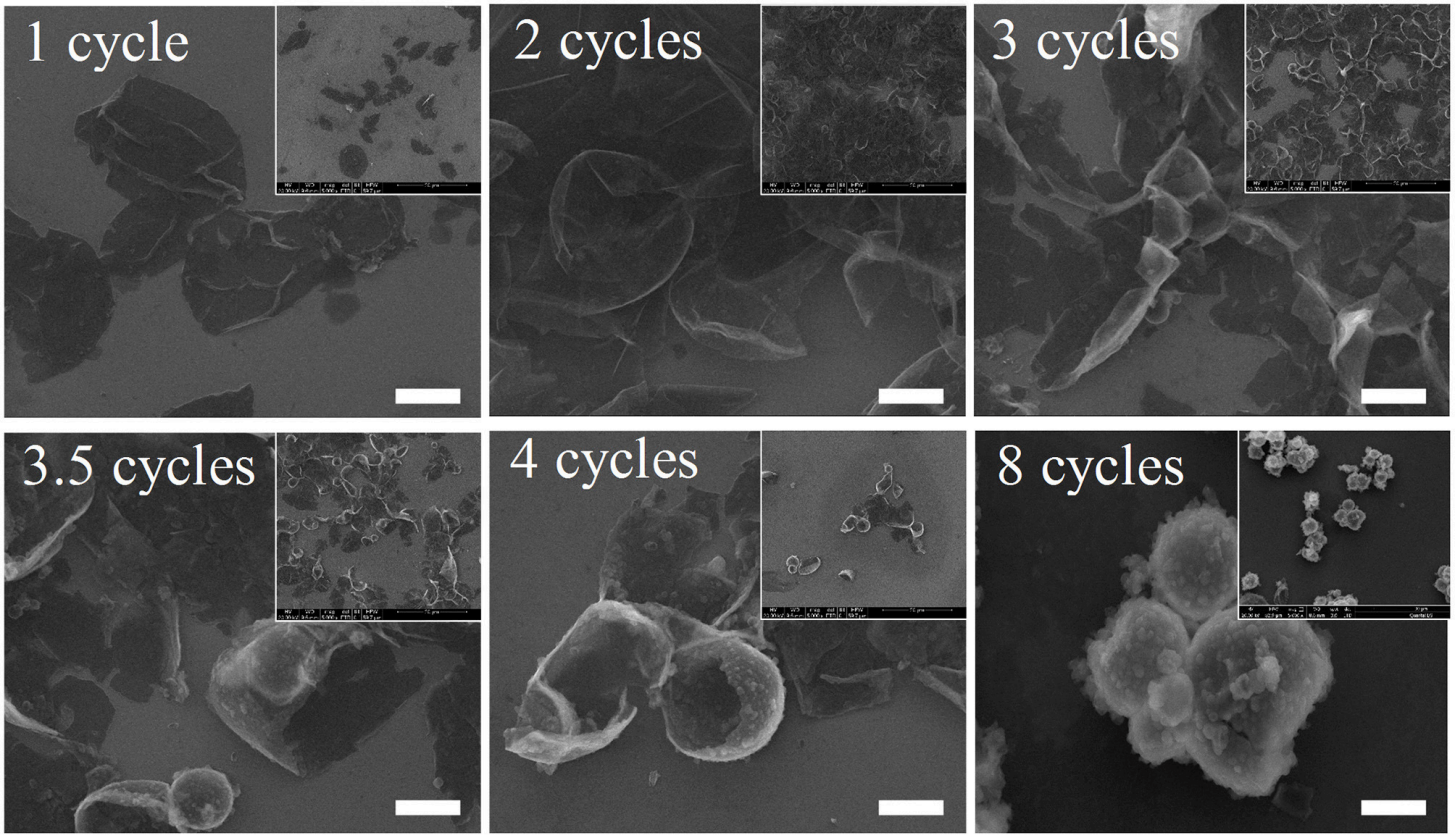
Scanning electron microscopy images of 1,3,5-trimethylbenzene-loaded MCs prepared with different numbers of deposition cycles. Scale bars represent 2 μm.

The MCs deposited for different cycles also showed differences in physical stability. Figure 1 shows the scanning electron microscopy (SEM) images of the 1,3,5-trimethylbenzene-loaded MCs prepared with different numbers of deposition cycles. As depicted in the images, all of the samples had an average size of approximately 3 μm. It is apparent that no MCs deposited for 1 cycle could preserve a relatively stable spherical shape. Additionally, apparent folds and creases could be observed for nearly all collapsed MCs. The membrane thickness and strength are prone to increase with sequential deposition, although no significant improvement was observed until deposition for 4 cycles. Fortunately, nearly all MCs can retain stable spherical shapes after we implemented 8 deposition cycles, even though aggregates composed of 2–6 MCs were prevalent in the sample. This also confirmed our aforementioned hypothesis about the instability of the system with increased deposition cycles derived from the variation in the zeta potential. Furthermore, the membrane surfaces of the MCs were covered with small protuberances or particles, indicating a disordered deposition of wall materials.

**Figure 2:**
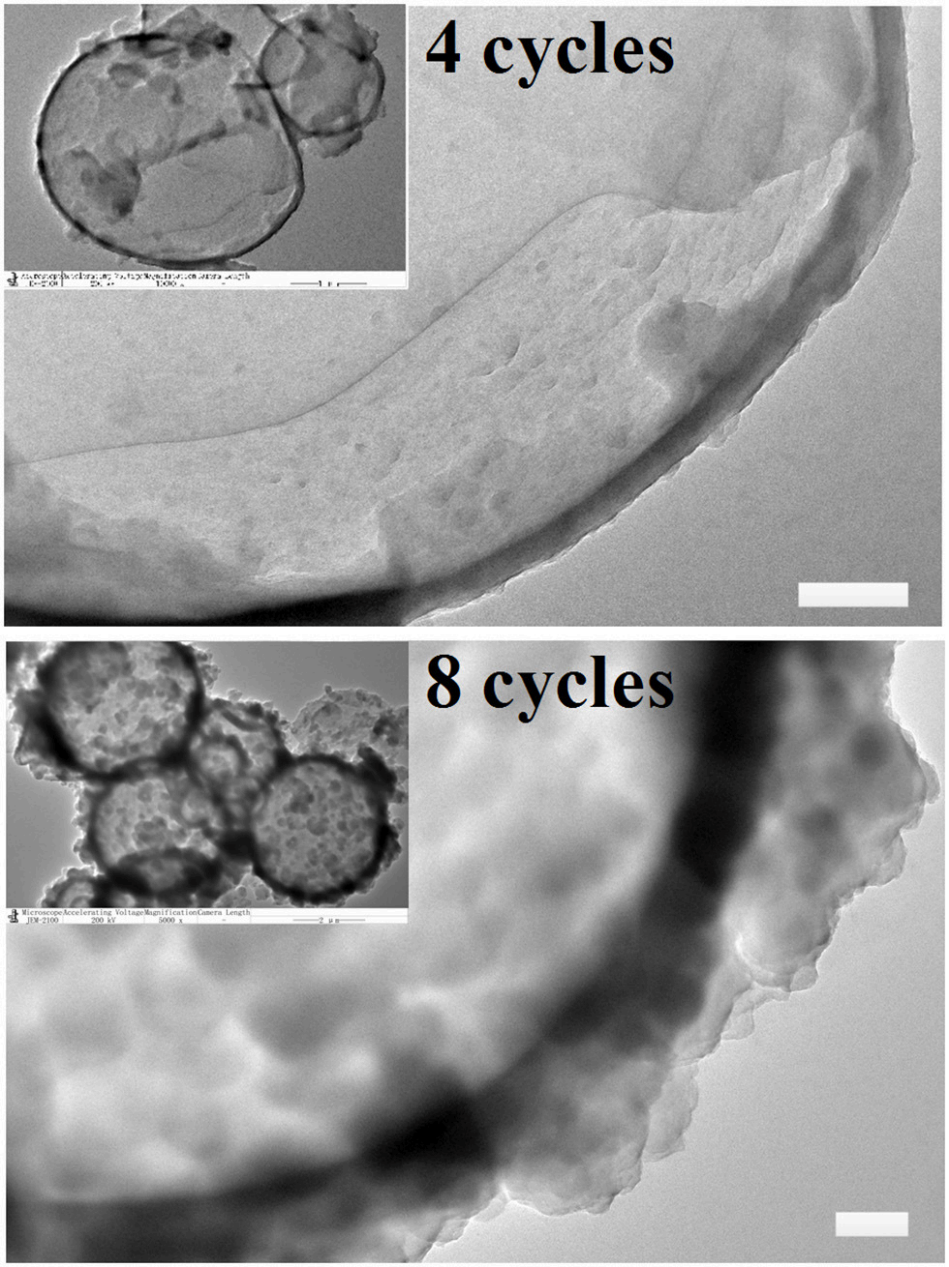
Typical transmission electron microscopy images of 1,3,5-trimethylbenzene-loaded MCs prepared with 4 and 8 deposition cycles. Scale bars represent 200 nm.

As was extensively reported, mechanical strength of MCs shows direct correlation with membrane thickness. In this research, membrane thickness and the morphology of MCs were determined by using TEM. SEM images showed that MCs may maintain stable shapes after deposition for 4 cycles, and we therefore selected MCs deposited for 4 and 8 cycles as a model for TEM observation. As illustrated in Figure 2, the surface of MCs deposited for 4 cycles was smooth, whereas that of the MCs deposited for 8 cycles appears rough and prone to agglutination, consistent with SEM images. In addition, many protuberances were observed in the TEM images of 8 deposition cycles. Fortunately, the membrane thickness of MCs could be roughly estimated. For twenty MCs, the membrane thicknesses of MCs deposited for 4 cycles ranged between 62 and 212 nm (128 ± 35 nm), whereas that for 8 cycles ranged between 183 and 492 nm (318 ± 87 nm). Clearly, the membrane thicknesses were much larger than that reported by Ejima et al. (2013) on polystyrene particles (membrane thickness was approximately 40 nm for MCs at 4 cycles). We suppose that the presence of calcium lignosulfonate in the current study greatly contributed in the membrane deposition process and therefore increased the membrane thickness of the MCs.

**Figure 3:**
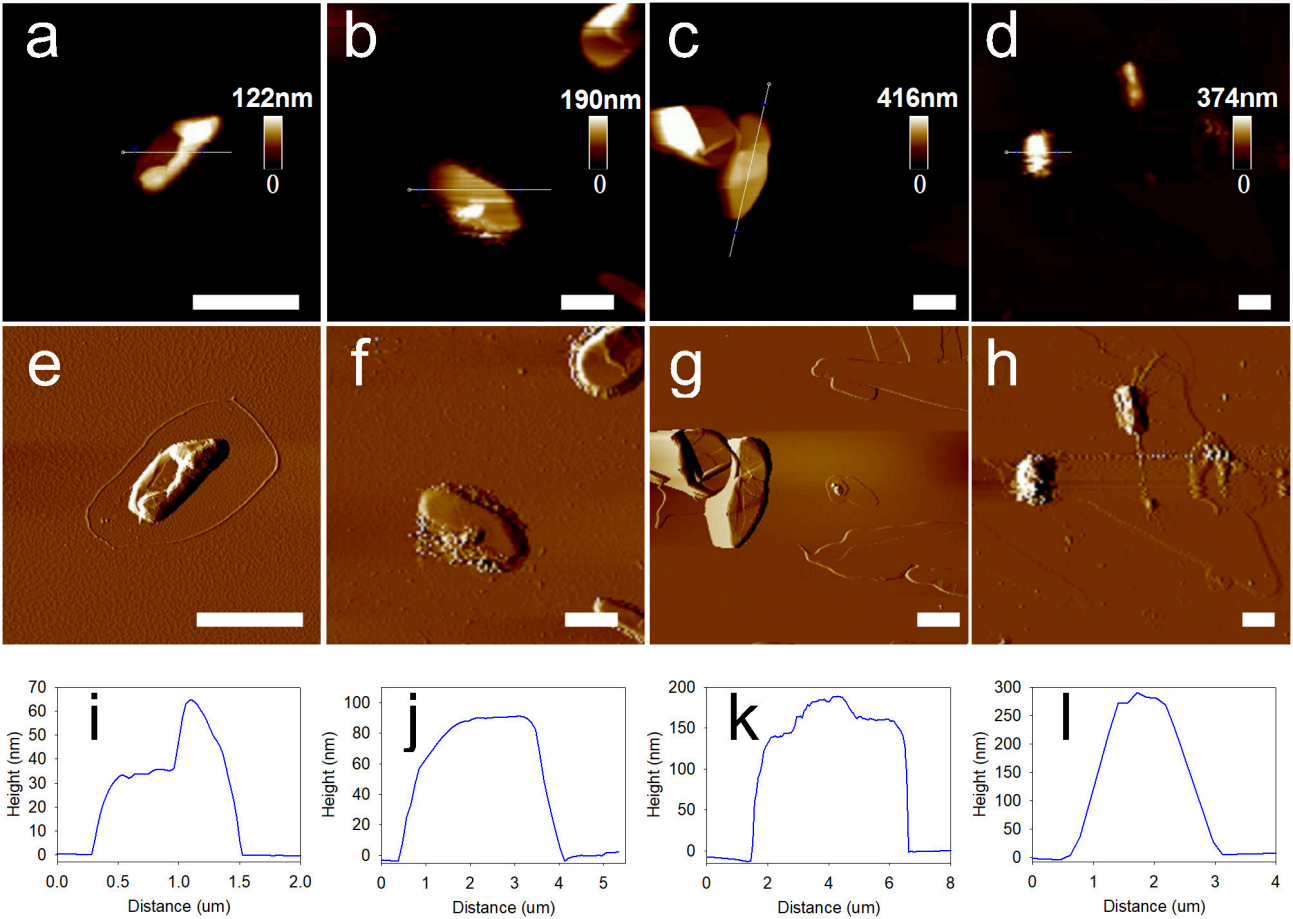
Morphology and membrane thickness of 1,3,5-trimethylbenzene-loaded MCs prepared with sequential deposition cycles by using Fe^3+^-TA system. **(a–d)** Typical AFM images of MCs deposited for 2, 4, 6, and 8 cycles. **(e–h)** Corresponding amplitude error images and **(i–l)** height analysis profiles of MCs prepared with different deposition cycles. Scale bars represent 2 μm.

Atomic force microscopy (AFM) was extensively reported to provide high-resolution simulated topographical images by meticulously analyzing surface height data. In the current study, methanol was used for dissolution of core materials in the MCs to yield hollow MCs before the AFM measurements. Consequently, typical collapses of the MCs were observed in all AFM images, as depicted in Figures 3a–d. Corresponding amplitude error images also indicated that the surface of 1,3,5-trimethylbenzene-loaded MCs was relatively smooth at 2 and 4 deposition cycles, while creases and rough surfaces were observed for MCs with 6 and 8 deposition cycles (Figures 3e–h). Then, relatively flat regions of the collapsed MCs in the AFM images were selected to determine the average membrane thicknesses of the MCs. Figures 3i–l illustrate the representative height analysis profiles of MCs prepared with different numbers of deposition cycles. Membrane thicknesses of 1,3,5-trimethylbenzene-loaded MCs were measured by examining 20 MCs by AFM height analysis. The average membrane thicknesses of MCs prepared with 2, 4, 6 and 8 deposition cycles were determined as 31.3 ± 4.6, 92.4 ± 15.0, 175.4 ± 22.1 and 254.8 ± 24.0 nm, respectively.

**Figure 4:**
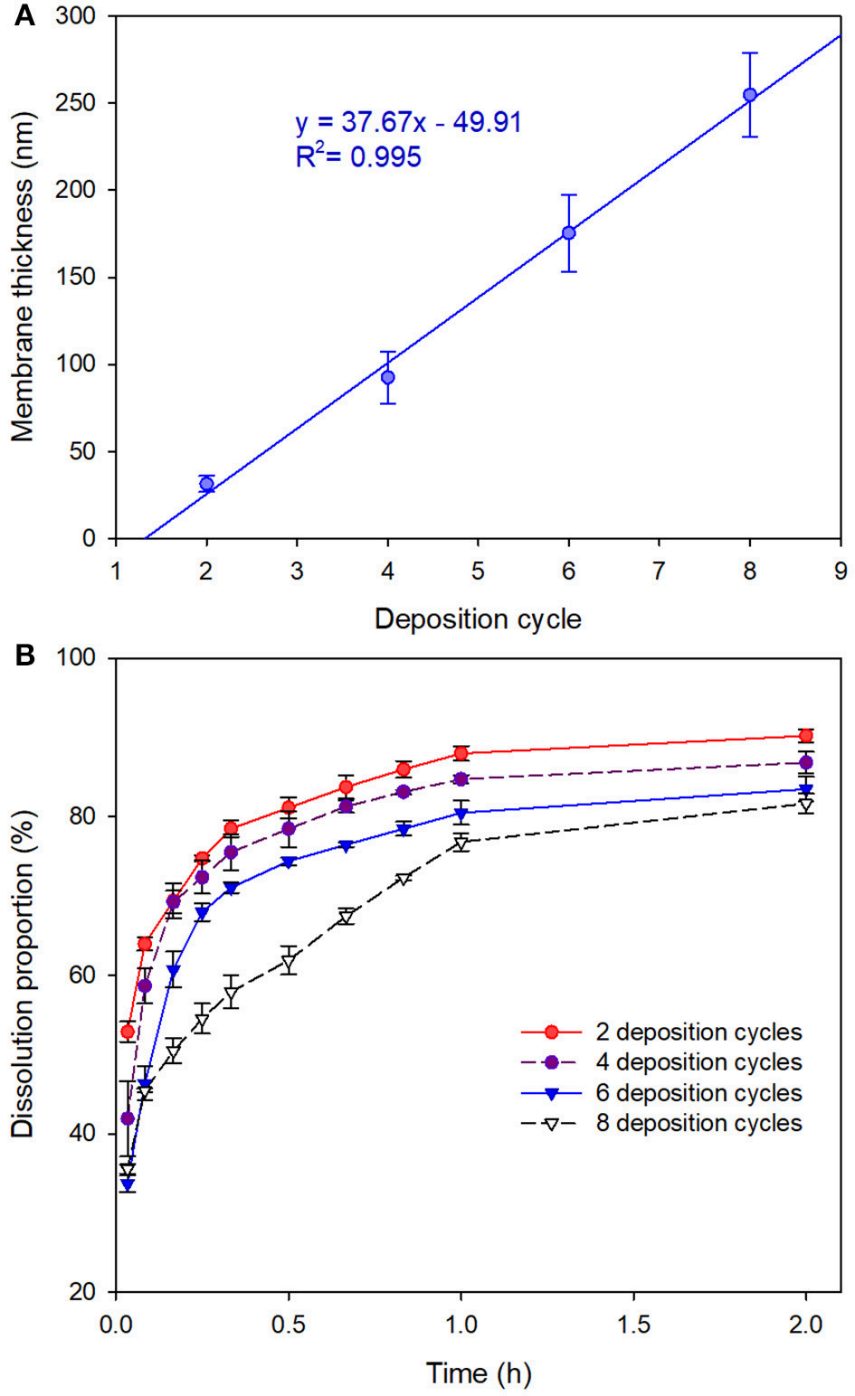
**(A)** Membrane thicknesses of 1,3,5-trimethylbenzene-loaded MCs (prepared with Fe^3+^-TA) by measuring 20 MCs via AFM height analysis. Data are represented as the mean ± SD. **(B)** Release profiles of the MCs in artificial release medium.

As depicted in Figure 4A, the membrane thickness of the MCs increased linearly with the deposition cycles, yielding a regression equation of *y* = 37.67x−49.91 (*R*^2^ = 0.995). We noticed that the membrane thickness values of the MCs measured by AFM were slightly inconsistent with the values obtained by TEM observation. We attributed the inconsistence to the tiny discrepancy in the sampling process. As we have seen in the SEM and TEM images, a certain amount of agglomerated MCs are present in the sample. However, we cannot measure the membrane thicknesses of agglomerated MCs due to the limitation of the AFM probe. As we had selected tapping mode for the AFM scans, the recommended AFM probe of the system was the RTESP probe. The cantilever of the probe has the thickness of 3.5–4.5 μm, length of 115–135 μm and width of 30–40 μm. The probe can hardly approach the surface of the agglomerated MCs because the height of the agglomerated MCs can easily exceed the thickness of the probe and thus leads to unsuccessful measurements. For a next-best option, we measured the thickness of MCs with relatively better dispersibility. Therefore, membrane thicknesses of the MCs measured by AFM analysis were slightly smaller than those measured by TEM observation because the well-dispersed MCs show fewer or smaller protuberances. In addition, the existence of the protuberances might also be the cause of higher standard deviations of the membrane thicknesses of MCs with larger deposition cycles (Figure 4A).

Figure 4B displays the release profiles of the MCs in artificial release medium. Although initial burst releases were observed for all the tested MCs, sequential deposition revealed significant advantages in controlled-release of the core material. The MCs with 8 deposition cycles released 35.5 ± 0.6% of the active ingredient in initial burst stage, whereas those with 2 deposition cycles dissolved 52.9 ± 1.3% of the active ingredient. Subsequently, the dissolution data of the core material vs. time were submitted to fit with zero-order, first-order and Higuchi models. The modeling results suggested that the release profiles of all the four tested MCs followed Higuchi kinetics as they yielded the highest determination coefficients (Table S2).

### Preparation and characterization of MCs prepared using Ca^2+^ and TA

**Figure 5:**
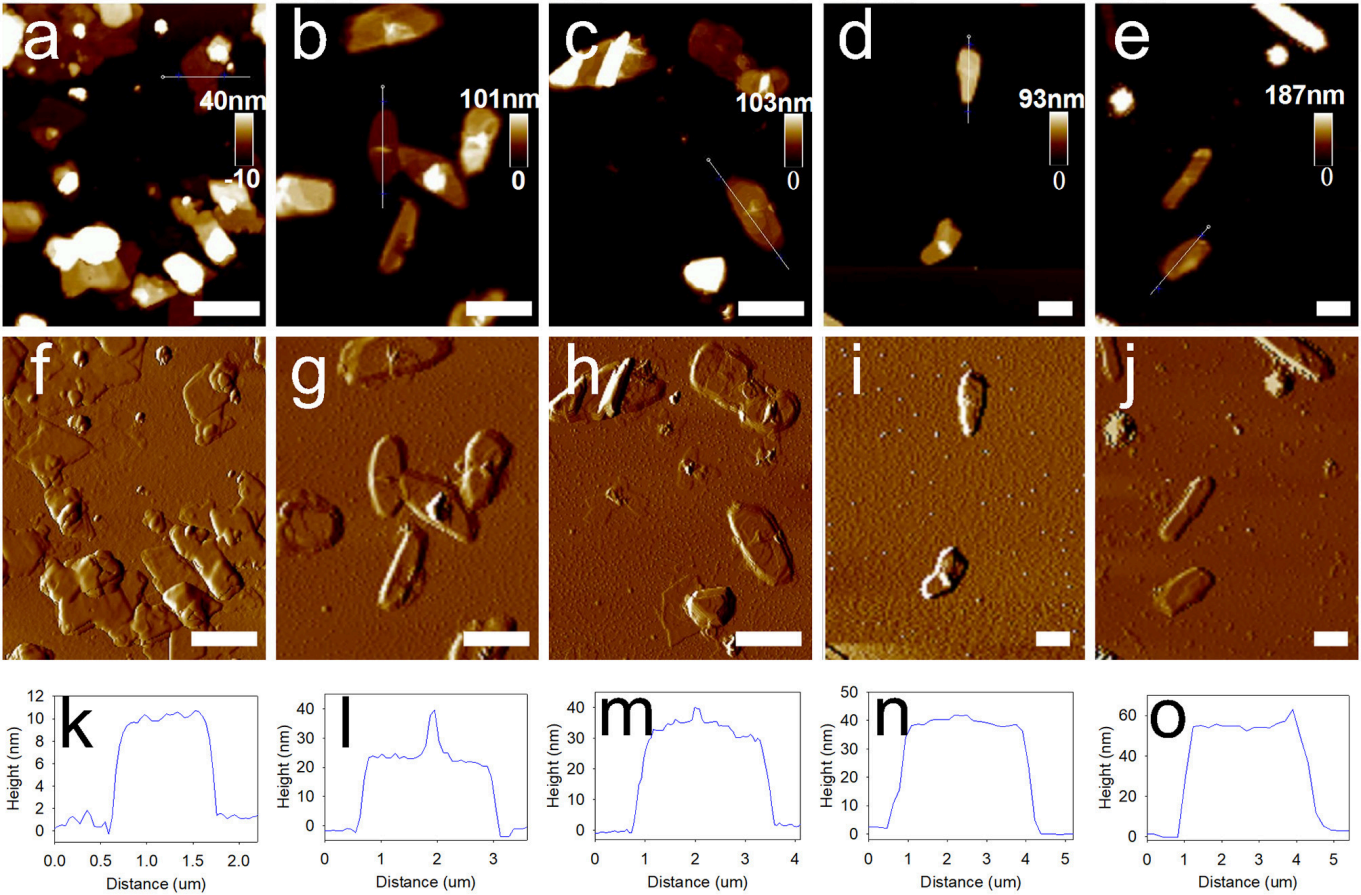
Morphology and membrane thickness of 1,3,5-trimethylbenzene-loaded MCs prepared with sequential deposition cycles by using Ca^2+^-TA system. **(a–e)** Typical AFM images of MCs deposited for 1, 2, 3, 4, and 5 cycles. **(f–j)** Corresponding amplitude error images, and **(k–o)** height analysis profiles of MCs prepared with different deposition cycles. Scale bars represent 2 μm.

We had also preliminarily investigated whether the type of metal ions influenced the deposition process. Thus, we used the Ca^2+^-TA network for the deposition on the solvent-water interface. Subsequently, we obtained a series of MCs prepared with Ca^2+^-TA by simply replacing Fe^3+^ with Ca^2+^ in the system. AFM height analysis images (Figures 5a–e) and amplitude error images (Figures 5f–j) indicate that 1,3,5-Ttrimethylbenzene-loaded MCs deposited with Ca^2+^-TA were much smoother than those prepared using the Fe^3+^-TA system. Corresponding representative height analysis profiles of MCs prepared with different numbers of deposition cycles are depicted in Figures 5k–o. Membrane thicknesses of the MCs were also measured by examining 20 MCs for each sample. The average membrane thicknesses of MCs prepared with 1, 2, 3, 4 and 5 deposition cycles were determined as 10.0 ± 1.1, 22.0 ± 2.5, 30.5 ± 3.3, 43.0 ± 4.5 and 59.7 ± 7.3 nm, respectively. As shown in Figure S1, the membrane thickness of the MCs also increased linearly with the sequential deposition cycles, as characterized by the regression equation of *y* = 12.02x−3.043 (*R*^2^ = 0.986).

## Conclusions

The common solvent 1,3,5-trimethylbenzene was used as a model solvent for encapsulation using coordination assembly between metal ions and tannic acid. Zeta potential, energy dispersive spectroscopy and X-ray photoelectron spectroscopy demonstrated the deposition of Fe^3+^-TA complexes on the solvent-water interface. According to atomic force microscopy height analysis, membrane thickness of the MCs increased linearly after sequential deposition. We can easily tune the membrane thickness of the MCs by adjusting metal ions or deposition cycles according to the application requirements.

## Author contributions

BL designed all the experiments, performed the preparation of the microcapsules and wrote the manuscript. XL and YL performed the SEM and TEM measurements. DZ and JL performed the AFM measurement and dissolution test. WM and FL supervised all the experiments and revised the manuscript.

### Conflict of interest statement

The authors declare that the research was conducted in the absence of any commercial or financial relationships that could be construed as a potential conflict of interest.

## Supplementary material

Table S1 Detailed information about the element composition of representative 1,3,5-trimethylbenzene-loaded MCs is listed in this table.

Table S2 Displays the fitness of the release profiles of 1,3,5-trimethylbenzene-loaded MCs to different models.

Figure S1 Shows the zeta potential of samples prepared with different deposition cycles.

Figure S2 Displays the energy dispersive spectroscopy image and X-ray photoelectron spectroscopy spectra of 1,3,5-trimethylbenzene-loaded MCs.

Figure S3 Describes the membrane thicknesses of 1,3,5-Trimethylbenzene-loaded MCs (prepared with Ca^2+^-TA) by measuring 20 MCs via AFMheight analysis.
