# Supplementary material for: Easily Tunable Membrane Thickness of Microcapsules by Using a Coordination Assembly on the Liquid-Liquid Interface
Source: Front Chem. 2018 Sep 7;6:387. doi: 10.3389/fchem.2018.00387 (PMC6137620; doi:10.3389/fchem.2018.00387)
Supplement: Table S1 — Detailed information about the element composition of representative 1,3,5-trimethylbenzene-loaded MCs is listed in this table. [file Table_1.docx]

**Supplementary material**

**Easily tunable membrane thickness of microcapsules by using a coordination assembly on the liquid-liquid interface**

Bei-xing Li,^1,2^ Xiao-xu Li,^1^ Yang Liu,^1^ Da-xia Zhang,^1,2^ Jin Lin,^2^ Wei Mu,^2^ Feng Liu,^1,2*^

1. Key Laboratory of Pesticide Toxicology & Application Technique, College of Plant Protection, Shandong Agricultural University, Tai’an, Shandong 271018, P. R. China

2. Research Center of Pesticide Environmental Toxicology, Shandong Agricultural University, Tai’an, Shandong 271018, China

^*^Corresponding author: Feng Liu, Professor, Tel: +86-0538-8242611, E-mail: fliu@sdau.edu.cn, College of Plant Protection, Shandong Agricultural University, 61 Daizong Street, Tai’an, Shandong 271018, P.R. China

**Table S1** Element composition of representative 1,3,5-trimethylbenzene-loaded MCs.

| Element | Standard sample | Weight % | atom % |
| --- | --- | --- | --- |
| C | CaCO_3_ | 51.28 | 61.32 |
| O | SiO_2_ | 40.44 | 36.30 |
| S | FeS_2_ | 0.28 | 0.13 |
| Cl | KCl | 1.00 | 0.41 |
| Ca | Wollastonite | 0.47 | 0.17 |
| Fe | Fe | 6.52 | 1.68 |

Note: The 1,3,5-trimethylbenzene-loaded MCs deposited for 3.5 cycles were selected as a model.

**Table S2** Fitness of the release profiles of 1,3,5-trimethylbenzene-loaded MCs to different models.

| Deposition cycle | Model | Empirical equation | *R*^2^ |
| --- | --- | --- | --- |
| 2 | Zero-order | Q_t_ = 15.55t + 67.71 | 0.6080 |
|  | First-order | Ln(1- Q_t_) = −0.7485t – 1.136 | 0.7862 |
|  | Higuchi | Q_t_ = 29.02t^1/2^ + 57.08 | 0.8264 |
| 4 | Zero-order | Q_t_ = 16.69t + 63.40 | 0.5099 |
|  | First-order | Ln(1- Q_t_) = −0.6497t – 1.038 | 0.6944 |
|  | Higuchi | Q_t_ = 32.059t^1/2^ + 51.37 | 0.7349 |
| 6 | Zero-order | Q_t_ = 19.62t + 55.78 | 0.5235 |
|  | First-order | Ln(1- Q_t_) = −0.6226t − 0.8467 | 0.6883 |
|  | Higuchi | Q_t_ = 37.60t^1/2^ + 41.69 | 0.7510 |
| 8 | Zero-order | Q_t_ = 21.62t + 47.70 | 0.7788 |
|  | First-order | Ln(1- Q_t_) = −0.6320t − 0.6216 | 0.8923 |
|  | Higuchi | Q_t_ = 38.02t^1/2^ + 34.50 | 0.9400 |

Note: Q_t_ is the dissolution proportion of 1,3,5-trimethylbenzene at time t.

**

**

**Figure S1** Zeta potential of samples prepared with different deposition cycles. Data displayed as the means ± SD (n = 3). Data with different lower-case letters are significantly different at p < 0.05 level according to Tukey’s test.

**
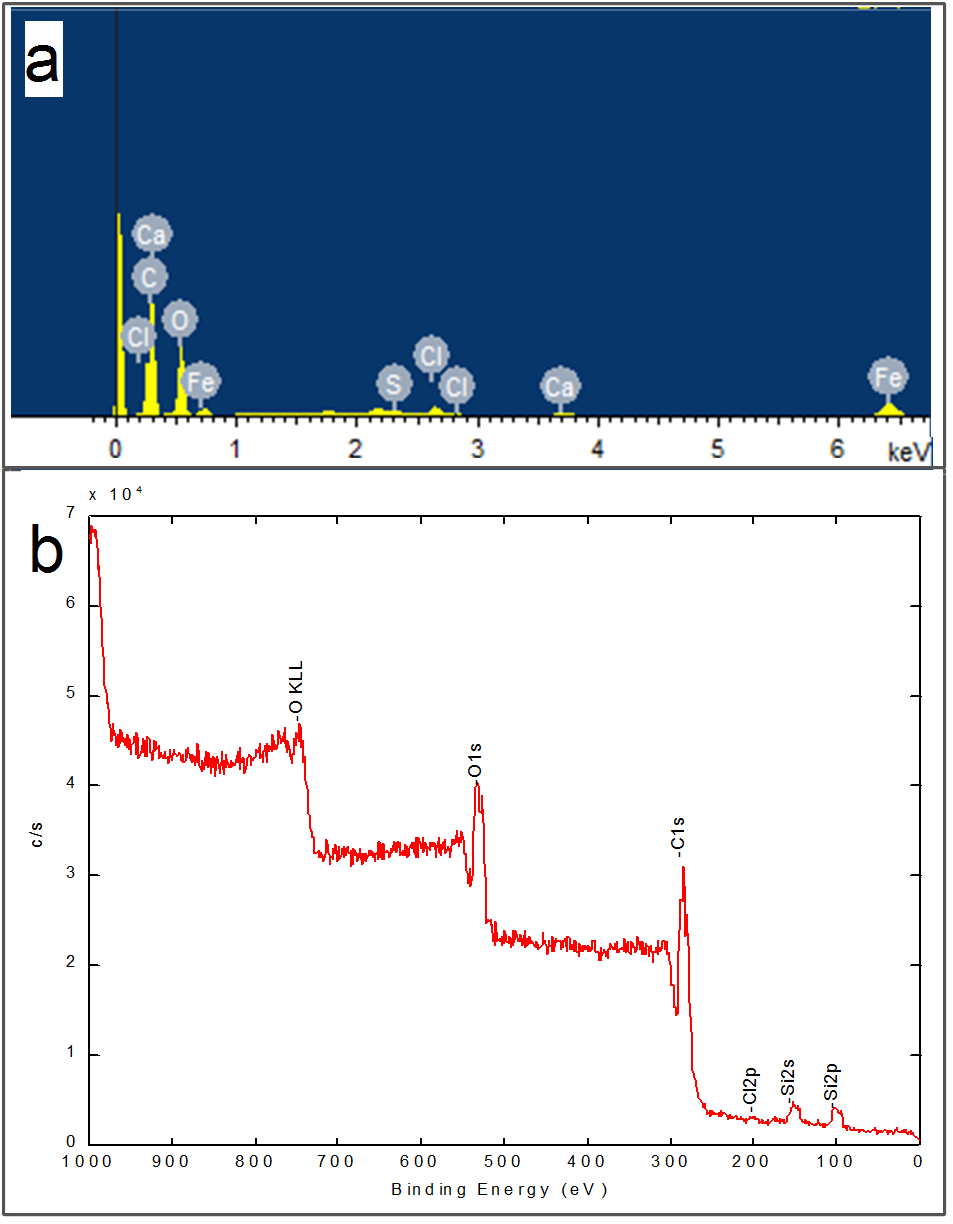
**

**Figure S2** (a) Energy dispersive spectroscopy confirms the presence of TA and Fe in the membrane. (b) X-ray photoelectron spectroscopy spectra of 1,3,5-trimethylbenzene-loaded MCs.

**

**

**Figure S3** Membrane thicknesses of 1,3,5-Trimethylbenzene-loaded MCs (prepared with Ca^2+^-TA) by measuring 20 MCs via AFM height analysis. Data are represented as the mean ± SD.
